# Feeling a Little Blue After Ablation: Iatrogenic Atrial Septal Defect With Right-to-Left Shunt Following Atrial Fibrillation Ablation

**DOI:** 10.7759/cureus.62629

**Published:** 2024-06-18

**Authors:** Lindsey Nguyen, Andrew Arbogast, Hayden Ivey, Amanda Frugoli, Jonathan Dukes

**Affiliations:** 1 Medicine, Community Memorial Hospital, Ventura, USA; 2 Internal Medicine, Community Memorial Hospital, Ventura, USA; 3 Electrophysiology, Community Memorial Hospital, Ventura, USA

**Keywords:** cryotherapy and laser cardiac ablation, intracardiac shunting, iatrogenic atrial septal defect, atrial septal defect, atrial fibrillation

## Abstract

Atrial fibrillation is the most common cardiac arrhythmia. Cardiac ablation is indicated for patients refractory to medical management. During the ablation process, a transseptal puncture is utilized to access and isolate the pulmonary veins, which results in a temporary iatrogenic atrial septal defect (iASD). Generation of an iASD is considered unavoidable and is a generally accepted risk due to high rates of spontaneous closure. Studies have shown that persisting iASD may occur in 5%-20% of patients for up to nine to 12 months after undergoing radiofrequency ablation and that spontaneous rates of closure are high in patients with normal intracardiac pressures.

Patients with preexisting elevated right intracardiac pressures from pulmonary hypertension or other right-sided cardiac pathology are at an increased risk of complications from iASD. These increased pressures can lead to clinically significant hypoxemia from right-to-left shunting following a transseptal puncture. Intervention with closure is considered in high-risk settings such as right atrial or ventricular enlargement, right-to-left shunting with hypoxemia, and intraseptal defect greater than 8 mm.

This case vignette describes a 67-year-old female who developed clinically significant right-to-left shunting intraoperatively from iASD with ongoing hypoxemia for several months but with spontaneous closure. We highlight this case as it demonstrates spontaneous closure in a high-risk iASD. We also provide a review of the literature on iASD after cardiac ablations.

## Introduction

Atrial fibrillation (Afib) is the most common cardiac arrhythmia, with an estimated prevalence between 2.7 and 6.1 million in the United States [1]. The incidence is anticipated to continue to increase, reaching up to 12.1 million by 2030 [1]. It is more common in people greater than 60 years old, with a greater incidence in males, although this appears to even out with advancing age. 

Studies have revealed that 80% of the aberrant Afib foci originate in the pulmonary veins [2,3]. Other, less common sites for ectopic foci include the superior vena cava (SVC) and the coronary sinus [2,3]. Medical management of Afib generally consists of pharmacological ventricular rate or rhythm control and stroke prophylaxis in appropriate patients. When pharmacological therapies fail, or when symptoms impair quality of life, catheter ablation is an appropriate treatment [2,4,5]. A recent randomized controlled study, *The Catheter Ablation Versus Anti-arrhythmic Drug Therapy for Atrial Fibrillation*, or the CABANA trial, found that those who underwent Afib ablation were less likely to have recurrence compared to those only treated with pharmacotherapy (50% vs. 69%) [5,6,7]. During the ablation procedure, catheters equipped with either radiofrequency, cryoballoon, or laser, cauterize the source of aberrant electrical signals near the pulmonary veins. This scars the cardiac tissue, thus blocking the transmission of electrical impulses to the rest of the atria and preventing Afib.

During the procedure, a catheter is inserted into the femoral vein and advanced through the venous system to the right atria. The catheter pierces through the atrial wall at the level of the fossa ovalis and creates a small atrial septal defect to access the left atrium and pulmonary veins [8]. In radiofrequency ablation or laser ablation, the catheter directly contacts the cardiac tissue before the high-energy waves cauterize the aberrant focus [8,9,10]. In cryoballoon methodologies, a balloon is inflated within the ostium of the pulmonary arteries, allowing liquid nitrogen to freeze the myocardial tissue, leading to scar formation. This scar tissue prevents the transmission of electrical impulses.

Afib ablation has an efficacy of around 62.3% after a single procedure at five years [8]. This number increases up to 79% at five years if a second follow-up procedure is performed. Based on current evidence, there is no difference in outcome between the types of ablation modalities, but laser ablations are less common [9,11]. Potential complications of ablation include vascular damage such as AV fistula and pseudoaneurysm, stroke, pulmonary vein stenosis, and structural heart damage, leading to heart block. Atrial septal puncture is a necessary step in the procedure, and persistent ASD may occur in 5%-20% of patients nine to 12 months after the procedure [9].

Patients undergoing cardiac ablation for treatment of Afib may be at risk for right-to-left shunting following transseptal puncture. This can occur when a concomitant right-sided cardiac pathology causes elevated right intracardiac pressures. In this case vignette, we describe a rare case of persistent atrial septal defect following ablation and acute onset of right-to-left shunting in a patient with paroxysmal Afib and heart failure with preserved ejection with preexisting elevated right intracardiac pressure. This case highlights a high-risk iatrogenic atrial septal defect (iASD) with spontaneous closure.

## Case presentation

**Figure 1:**
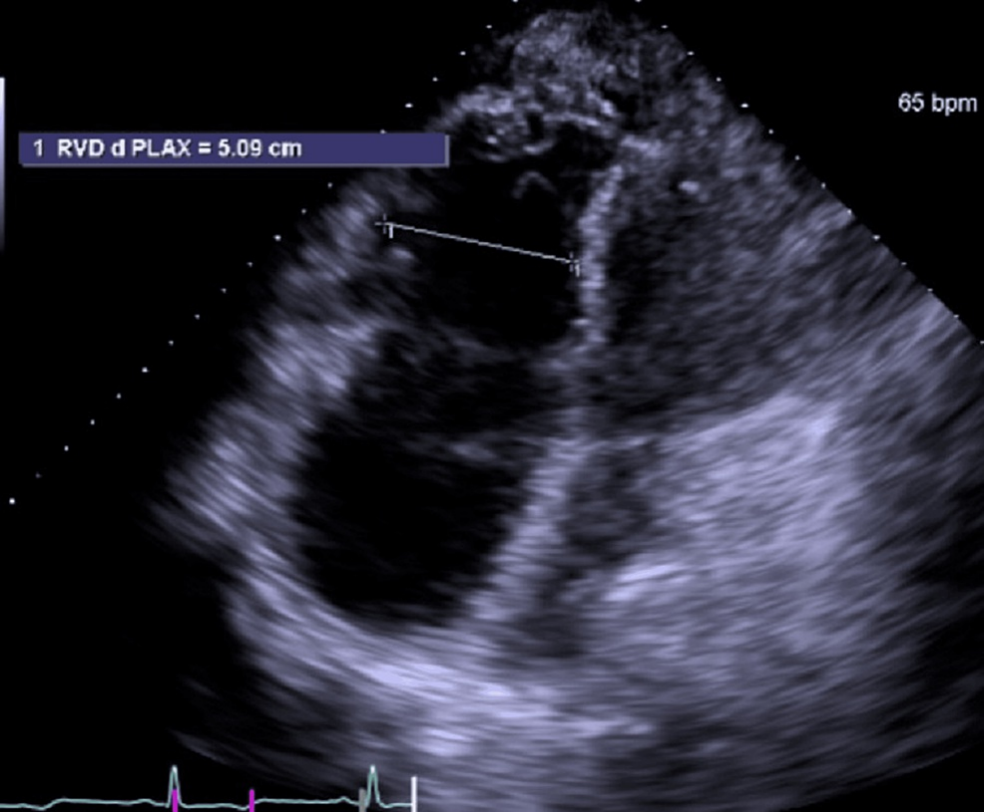
Preprocedure echocardiogram. RVD, right ventricle diameter

A 67-year-old female with hypertension, hyperlipidemia, heart failure with preserved ejection fraction (HFpEF), obstructive sleep apnea (OSA) noncompliant with continuous positive airway pressure (CPAP), and paroxysmal atrial fibrillation presented for an elective cardiac ablation. She had symptomatic paroxysmal atrial fibrillation and was unable to tolerate amiodarone. She continued to have symptomatic episodes despite treatment with metoprolol. She was on stroke prophylaxis with apixaban. Additionally, she had undergone a prior evaluation with an echocardiogram, which demonstrated a dilated right atrium with severe right ventricular enlargement, severe tricuspid regurgitation, and an estimated pulmonary artery pressure of 40 mmHg (Figure 1). She had normal left ventricular systolic function. Her elevated right heart pressures were suspected to have untreated OSA.

She was asymptomatic in sinus rhythm. She was provided conscious sedation for the procedure. Isolation of pulmonary veins and cavotricuspid isthmus was completed via a standard radiofrequency double transseptal puncture technique. She underwent successful ablation of the right and left pulmonary veins using a 3F navigation and an 8F radiofrequency ablation catheter. When switching to the second half of the procedure to ablate the cavotricuspid line, she became hypoxemic with oxygen saturation in the low 80s, requiring increasing FiO_2_ requirements to maintain oxygen saturation. Flash pulmonary edema was suspected, and she was given IV Furosemide. Postoperatively, she was transferred to the ICU and weaned off the mechanical ventilator but still required bilevel positive airway pressure (BiPAP) to maintain adequate oxygen saturation, with a FiO_2_ of 40% and positive end-expiratory pressure (PEEP) of 8 cm H_2_O. The next day, her FiO_2_ requirement increased to 55%.

Two days post-op, the patient was weaned from BiPAP but continued to require 5L of oxygen via nasal cannula. A two-dimensional (2D) echo with bubble contrast revealed an iASD with right-to-left shunting and a small amount of expected pericardial fluid (Video 1).

**Video 1:** Two-dimensional (2D) transthoracic echocardiogram demonstrating trivial pericardial effusion and persistent atrial septal defect with right-to-left shunting. iASD, iatrogenic atrial septal defect

After continued diuresis, there was a modest reduction in her oxygen support requirement. She was discharged home on a 4L nasal cannula with nightly CPAP and continued diuretics. Amiodarone was started to reduce the risk of Afib recurrence, and stroke prophylaxis with apixaban was continued. She had plans for a close cardiac follow-up in two weeks for evaluation and planning for repeated echocardiogram. Discussion regarding iASD closure was held, but conservative treatment and supportive care with close follow-up were chosen, as many iASDs have spontaneous closure. She also planned to have an outpatient evaluation with right and left cardiac catheterization to determine the etiology of her elevated right heart pressure.

Following hospital discharge, the patient's oxygenation status was successfully maintained on a 2L nasal cannula. A follow-up echo at two months revealed a persisting right-to-left shunt unchanged from discharge. At three months, an echo revealed spontaneous shunt closure and resolution of hypoxia.

## Discussion

Our patient's intraoperative hypoxia was suspected to be from pulmonary edema, but additional evaluation with transthoracic 2D echocardiogram revealed a small interatrial right-to-left shunt through the defect created by the transseptal puncture. Although the cause of her elevated right atrial and enlarged right ventricle will need further evaluation, they are the suspected risk factors for this complication. It is suspected that the patient had chronic elevated pressures from known OSA with chronic CPAP noncompliance and/or right heart failure secondary to left heart failure. We hypothesized that the chronic increased right-sided pressures resulted in a clinically significant shunt and prolonged hypoxemia.

Generation of an iASD is considered unavoidable for atrial fibrillation ablations. It is a generally accepted risk due to high rates of spontaneous closure. Studies have shown that persisting iASD may occur in 5% to 20% of patients for up to nine to 12 months after undergoing radiofrequency ablation and that spontaneous rates of closure are high in patients with normal intracardiac pressures [9,10].

In the largest prospective study, the EVITA study by Nagy et al. evaluated the incidence and natural history of iASD. They found that at three months of follow-up, iASD persisted in 18.1% of patients, and by one year, 82.4% of the defects spontaneously resolved [10]. The study concluded that the spontaneous closure rate was high and that no difference was observed between the type of ablation (cryoballoon versus radiofrequency) or access technique with single versus double puncture. It’s important to note that the study participants who displayed normal intracardiac pressures all had defects characterized by asymptomatic left-to-right shunting. In addition, the low interatrial pressure gradient allows for relatively rapid spontaneous resolution; therefore, interventional repair is not indicated. In contrast, in patients with right heart failure or pulmonary hypertension, clinically significant hypoxemia results from a right-to-left pressure gradient that is maintained throughout the cardiac cycle, which fits this clinical vignette. 

A study by Alkhouli et al. attempted to identify possible predictors of persistent iASD, as well as proposed an algorithm for surgical closure, including intraoperative defects >8 mm, large left-to-right shunt, right-to-left shunt with hypoxemia, and severe right ventricular dysfunction [11]. The study further suggests that when right-to-left shunting is detected, intraprocedural measurement of PaO_2_/FiO_2_ could be useful to determine the clinical significance of the shunt. In the cohort, 15% had persistent iASD beyond six months [11]. According to other studies, the size of the catheter sheath, prolonged procedure times, pulmonary arterial hypertension, and excessive catheter manipulation may also play a significant role. Overall, the catheter size correlates linearly with the risk of iASD development and time to closure.

Mugnai et al. studied 127 patients at a single institution between 2008 and 2012 who underwent transseptal catheterization for either radiofrequency ablation or cryoballoon ablation [12]. Although they found that iASD incidence was higher in the cryoballoon ablation group (22.2%) compared with the radiofrequency group (8.5%), they speculated that the difference between these groups is due to the larger sheath diameter, 15F compared to 8.5F, respectively [12]. For patients with cryoballoon ablation, 20% had persistent iASD at the one-year follow-up [12]. These results were similar to prior publications by Mugnai et al. Furthermore, a study by Rich et al. investigated cryoballoon ablation techniques and the influence of the point of entry on the persistence of iASDs [13]. The study posited that transseptal puncture through a site anterior to the inferior limbus would decrease the frequency of persistent iASDs. This would be accomplished by decreasing the angle needed for the catheter to reach the pulmonary veins, thus reducing penetrative force [13]. Approximation of the myocardium would also be enhanced due to the increased thickness of the myocardium at the area anterior to the inferior limbus. Results demonstrated that 100% of ablation sites through the foramen ovale had left-to-right shunting immediately post-op. In comparison, only 33% had active shunting when the point of entry was anterior to the inferior limbus [13].

The role of pulmonary hypertension may be the most significant factor in our case. A small study by Hammerstingl et al. showed a high incidence of right-to-left shunt found in six of the 27 patients studied [14]. They determined that increased preprocedural pulmonary artery pressure is associated with a higher incidence of right-to-left shunt; however, parameters in relation to the severity of shunting and resulting hypoxemia remain undefined. At present, it could be inferred that more severe disease and higher differences in interatrial pressure gradients can result in more aggressive, continuous flow that may impede the usual rapid healing process leading to prolonged hypoxemia and delayed spontaneous closure. In this regard, further research is warranted to determine if there is a pulmonary arterial pressure threshold at which pathologic shunting is expected and intervention is indicated.

The high-risk features and course of illness demonstrated in our case meet the criteria for surgical intervention as defined by Alkhouli et al. However, her course of recovery revealed that medical management and supportive oxygen therapy were sufficient and that spontaneous resolution could occur. Her time to spontaneous closure is similar to the existing literature. Discussion of oxygen management in iASDs with right-to-left shunting may be controversial because oxygen therapy under these circumstances typically yields minimal results due to the volume of shunted blood that remains unventilated. Larger shunt volumes from more significant septal defects can result in more profound hypoxemia, as observed in our case. However, the oxygenation status in our patient was successfully maintained without invasive supportive measures or surgical intervention. It could be proposed that the volume of shunted blood was at a threshold low enough to be overcome with supplemental oxygen therapy, suggesting that there may be a place for oxygen therapy in certain patients.

Studies regarding risks for the development of a persistent iASD are currently limited [15]. Due to the possibility of a right-to-left shunt in patients with persisting defects, a screening methodology implemented before procedures may show benefits to prevent these adverse outcomes. Pulmonary artery hypertension could be an indication for this screening tool, as this value can be easily obtained via pre-ablation echo. Another consideration would be the measurement of intraoperative right atrial pressure. We recommend that further studies be conducted to evaluate the combined risk factors involving patient comorbidities and preoperative assessment. The development of a scoring system for risk determination can potentially optimize candidate selection and guide intraoperative intervention and postoperative management. 

## Conclusions

Cardiac ablation for the treatment of Afib is a safe and well-established treatment option for patients refractory to pharmacological management. A transseptal puncture is an integral step in the procedure that allows access to the left atrium to isolate the pulmonary veins. In patients with normal intracardiac pressures, some degree of left-to-right shunting is expected; however, persistent defects can occur. The most significant risk factor for persistent iASD development is catheter size. In patients who develop right-to-left shunting with significant hypoxia, certain high-risk features should be considered for intraoperative repair. Fortunately, a majority will achieve spontaneous closure around 12 months. However, repair may be indicated if the persisting defect results in symptoms that cause significant disability, or if it fails to close with conservative measures. In patients with elevated right intracardiac pressures from pulmonary hypertension or other right heart failures, as demonstrated on echocardiogram, preoperative assessment, and risk determination via screening tool may be beneficial. Research is needed in this area.

Our case report highlights spontaneous closure with supportive measures despite right-to-left shunting and underlying elevated right heart pressures. Additionally, the spontaneous closure time is similar to prior studies with closure between three and 12 months. 

More research should be pursued to further specify criteria that warrant supportive versus surgical management such as the size of the defect, severity of pulmonary arterial hypertension, degree of shunting, severity of disability, and long-term outcomes.
